# Beyond Words: Creative Arts Therapies as Pathways to Cognitive and Mental Health in Later Life

**DOI:** 10.1093/geroni/igaf122.1806

**Published:** 2025-12-31

**Authors:** Shoshi Keisari

## Abstract

Creative arts therapies are rooted in spontaneity, imagination, and creativity. They allow individuals to move beyond predictable patterns, engaging in novel and often surprising interactions. By fostering active participation, creative arts therapies allow people to engage as productive contributors to their communities. The arts can also bring to life the embodied and sometimes muted aspects of human experience that cannot be fully captured through verbal expression alone. This symposium brings together interdisciplinary research exploring how playful interactions, drama therapy, participatory theater, and phototherapy contribute to psychological resilience and cognitive health in later life. The first presentation investigates the impact of playfulness and improvisational theater on cognitive functioning and social engagement of older adults. It demonstrates how brief playful interactions can enhance executive functioning and positive emotions, exploring the physiological mechanisms underlying these effects. The second presentation reports on an RCT study examining tele-drama therapy for older adults with constricted mobility, highlighting its role in fostering psychological well-being and reducing depressive symptoms. The third presentation explores Theater-Based Participatory-Action-Research with caregivers, illustrating how theatrical methods help women caring for spouses with dementia navigate evolving relational roles and strengthen their sense of agency. The fourth study delves into phototherapy interventions for older women, revealing how visual self-representation can serve as a means of redefining societal perceptions of the aging body. Together, these studies underscore the significance of arts in aging research and practice. The discussion highlights practical applications for integrating these approaches into interventions that promote cognitive functioning and mental health in aging. Mental Health Practice and Aging Interest Group Sponsored Symposium

